# Biomass and lipid induction strategies in microalgae for biofuel production and other applications

**DOI:** 10.1186/s12934-019-1228-4

**Published:** 2019-10-21

**Authors:** Hossein Alishah Aratboni, Nahid Rafiei, Raul Garcia-Granados, Abbas Alemzadeh, José Rubén Morones-Ramírez

**Affiliations:** 1 Facultad de Ciencias Químicas, Universidad Autónoma de Nuevo León, UANL, Av. Universidad s/n, CD. Universitaria, 66451 San Nicolás de los Garza, NL Mexico; 2 Centro de Investigación en Biotecnología y Nanotecnología, Facultad de Ciencias Químicas, Universidad Autónoma de Nuevo León, Parque de Investigación e Innovación Tecnológica, Km. 10 autopista al Aeropuerto Internacional Mariano Escobedo, 66629 Apodaca, Nuevo León Mexico; 3 Department of Crop Production and Plant Breeding, School of Agriculture, Shiraz University, Km. 12 Shiraz-Isfahan Highway, Bajgah Area, Shiraz, 71441-65186 Iran; 4 Facultad de Ciencias Biológicas, Universidad Autónoma de Nuevo León, UANL, Av. Universidad s/n, CD. Universitaria, 66451 San Nicolás de los Garza, NL Mexico

**Keywords:** Global warming, Biofuel, Microalgae, Nanoparticle, Metabolic genetic engineering, Lipid production

## Abstract

The use of fossil fuels has been strongly related to critical problems currently affecting society, such as: global warming, global greenhouse effects and pollution. These problems have affected the homeostasis of living organisms worldwide at an alarming rate. Due to this, it is imperative to look for alternatives to the use of fossil fuels and one of the relevant substitutes are biofuels. There are different types of biofuels (categories and generations) that have been previously explored, but recently, the use of microalgae has been strongly considered for the production of biofuels since they present a series of advantages over other biofuel production sources: (a) they don’t need arable land to grow and therefore do not compete with food crops (like biofuels produced from corn, sugar cane and other plants) and; (b) they exhibit rapid biomass production containing high oil contents, at least 15 to 20 times higher than land based oleaginous crops. Hence, these unicellular photosynthetic microorganisms have received great attention from researches to use them in the large-scale production of biofuels. However, one disadvantage of using microalgae is the high economic cost due to the low-yields of lipid content in the microalgae biomass. Thus, development of different methods to enhance microalgae biomass, as well as lipid content in the microalgae cells, would lead to the development of a sustainable low-cost process to produce biofuels. Within the last 10 years, many studies have reported different methods and strategies to induce lipid production to obtain higher lipid accumulation in the biomass of microalgae cells; however, there is not a comprehensive review in the literature that highlights, compares and discusses these strategies. Here, we review these strategies which include modulating light intensity in cultures, controlling and varying CO_2_ levels and temperature, inducing nutrient starvation in the culture, the implementation of stress by incorporating heavy metal or inducing a high salinity condition, and the use of metabolic and genetic engineering techniques coupled with nanotechnology.

## Introduction

Recent reports from the Department of Economic and Social Affairs (DESA) show that the global human population is growing at alarming rates, predicting the world population will be higher than 9.8 billion in 2050 if the current population growth rate is maintained [1]. Therefore, within the next 30 years, society will face a series of problems that will put at risk the existence of most living organisms in the planet, and these problems include: energy crisis, global warming, greenhouse effects, toxic gases emission and drastic climate changes. This has created a concern in international scientific communities that seek to tackle one of the main sources of these problems: the use of fossil fuels as our main energy source [2]. One way to counteract the problem, is to seek an environmentally-friendly substitute of fossil fuels capable of satisfying the growing global energy demand [3]. Among the alternatives to fossil fuels, biofuels present relevant environmental advantages over the other options [4].

Biofuels are a renewable and sustainable alternative energy source; different regions of the world have used them to partially replace the use of fossil fuels, such is the case of Brazil with sugarcane, Europe and parts of Asia using mostly palm oil as their production source and for the case of biofuels produced from microalgae, countries like Brazil, Japan, China and the Unites States are the leaders in the field. Biofuel production is an important source of job creation, and the first generation of biofuels has allowed significant increments in farmers’ incomes [5–7]. These reasons propelled biofuels as a suitable alternative to fossil fuels [2, 8–10], especially for the automotive industry, which is one of the activities that generates the largest amounts of carbon dioxide worldwide [11].

Currently, a high percentage of the biofuels produced worldwide come from different raw organic materials, grouped into three different categories or generations [2, 12]. First-generation biofuels include those based on feedstocks that can be used for human consumption, including crops like maize, sugarcane, palm oil, sugar beet and wheat. Second-generation biofuels are those obtained from lignocellulosic feedstock, the non-edible parts from food crops that are usually discarded such as stems, leaves and husks [13–15]. Although, these generations of biofuels can partially satisfy the global energy demand, they depend on cultivable land available, being its main disadvantage; since cultivable land is limited and the space necessary for their production compete with the production of food crops intended for human consumption. Therefore, biofuels derived from edible or non-edible crops are not considered as the optimal alternative to fossil fuels [16]. A proposed solution to tackle these drawbacks is the third generation of biofuels, obtained from the cultivation of microalgae, unicellular photosynthetic microorganisms capable of converting CO_2_ and light into biomass and high-energy lipids, precursors of biofuels [17]. Compared with the first two generations of biofuels, the third generation have certain advantageous characteristics: they do not compete with food crop production or available farmland, they require less water, a higher CO_2_ mitigation rate, the potential to obtain nutrient sources from wastewater, higher carbon uptake and higher lipid content, at least 15–20 times higher than the second generation biofuels obtained from oleaginous crops [18–27]. Microalgae have rapid growth rates in favorable conditions, being able to generate a higher biomass production rate compared with land crops [28]. In addition, the space where microalgae can be cultivated is much smaller, a great advantage over other biofuel alternatives [29], and they are also capable of growing in wastewater or reject water, saline/brackish water and even sewage [30–32]. The use of biofuels obtained from microalgae have the possibility of reducing greenhouse gas effects as they are accountable for 40% of global carbon fixation and can reach up to 70% of oil content by dry weight in some strains [4]. Moreover, microalgae as a photosynthetic organism, use water and atmospheric CO_2_ to convert in a very efficient way, sunlight into chemical energy to produce from the carbon in CO_2_, valuable organic components such as proteins, carbohydrates and lipids [33, 34].

During the process of photosynthesis, nonpolar lipids like triacylglycerol (TAG), end up being stored in the microalgal cells [33]. It has been widely accepted that the production of these lipids serves as energy storage to microalgae cells. Despite this, they are valuable compounds since have an important commercial value [33, 35–37]. Through the process of trans esterification, the TAGs can be easily converted into fatty acid methyl esters which are an important and versatile form of biodiesel and the cornerstone for its production [2]. The best way to produce high amounts of these lipids is through the efficient large-scale cultivation of microalgae [38]. However, wild-type microalgae under environmental conditions are not capable of producing enough lipids to satisfy the global energy demands. Therefore, different techniques and approaches to enhance higher production rates of lipids in microalgae and make this process sustainable and scalable have been explored [2].

It is important to mention that the production of lipids in microalgal cells goes beyond energy storage since they use these lipids to construct their cellular membranes and are fundamental in the production of other biomolecules [2, 39]. Within recent years, researchers have been focused in studying different methods to increase the production of lipids in microalgae cells at different levels (induction with molecules, presence or absence of a factor, and specific growing/production conditions). Here we provide a more comprehensive review of the different lipid induction strategies in microalgae cells and their applications in biofuel production.

## Lipids in microalgae

Lipids produced by microalgae can be divided into two main groups: polar lipids, like glycerophospholipids, which have an important role in cell structure; and non-polar lipids, like triacylglycerols, mainly responsible for energy storage. Structural lipids (polar lipids) usually have long chains of fatty acids which could be transformed to obtain polyunsaturated fatty acids (PUFAs), this type of fatty acid includes Eicosapentaenoic acid (EPA), docosapentaenoic acid (DPA) and docosahexaenoic acid (DHA). PUFAs play an important role in the formation of mitochondrial super complexes [40], they have potential for biofuel production and have been found to have applications in the treatment of some diseases such as atherosclerosis, Parkinson and Alzheimer [41]. Polar lipids and some sterols provide a selectively permeable barrier that protects the cell from the outside and helps in the separation of the different intracellular organelles [42]. These lipids have special roles in the optimal maintenance of membrane fluidity for a variety of metabolic and biosynthetic processes and participate directly in different intracellular membrane fusion events. Moreover, these structural lipids have a significant function in cell signaling pathways and play a key role in response to changes in cellular environment [34, 42].

**Fig. 1:**
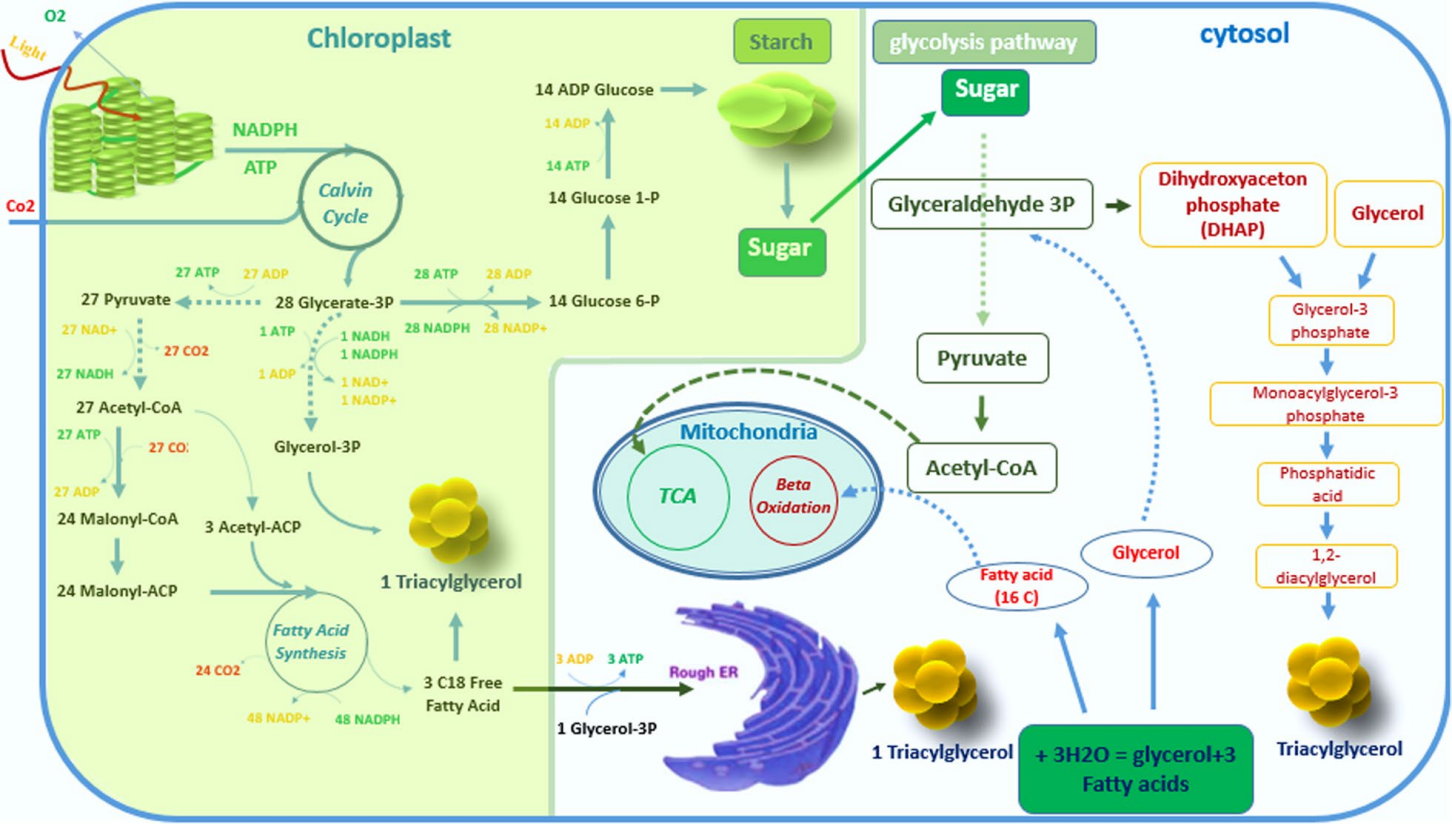
Simplified photosynthesis process and the three main possible biochemical pathways for TAGs formation: in the chloroplasts, in the ER, or in the cytosol

On the other hand, TAGs play a fundamental role in energy storage within the microalgae cell (Fig. 1), where the photosynthesis process generates basic energy by transforming sunlight into a useful molecule for the cell. To do this, cells use a molecule with a carbon skeleton (Glycerate-3P) and then convert it into more important molecules (such as pyruvate, glucose, xylose, acetate, amino acids, lipids, etc.); this very complex process helps the cell generate the biochemistry which is part of basal and complex metabolism, growth, energy storage and maintenance [43]. Moreover, less than 10% of these compounds can be metabolized to produce fatty acids in the chloroplast [44, 45].

The last product of the fatty acids can be used to produce phosphatidic acid and diacylglycerol in the endoplasmic reticulum (ER) and in the chloroplast, whose participation is known to act at the cellular metabolism level. Due to a variety of conditions and elements, the accumulation of TAGs (specially in situations such as the lack of nutrients or stress environments) can be influenced. Some ER-derived diacylglycerol can be used to assemble TAG in ER [46–49]; these TAGs are principally formed in the light period in ER and stored there, they could be reused for polar lipid constructing in the darkness period [50].

Microalgae cells are constituted of saturated and monounsaturated fatty acids [51]. It was believe that the red algae *Porphyridium cruentum* was the only one that could accumulate PUFAs in TAGs [52], however, recent studies have discover that some species of green microalgae, such as *Parietochloris incisa* have better abilities to produce high amounts of omega-6, long-chain PUFA (n-6 LC-PUFA) [53, 54]. Other species, such as *Pavlova lutheri*, *Nannochloropsis oculata*, *Thalassiosira pseudonana*, and *Phaeodactylum tricornutum* could also accumulate TAGs but in lower levels [55, 56].

Different studies have shown that TAGs can be used to perform more activities besides energy storage activation. For example, they could have an indirect role in the reorganization of membrane in response to a sudden changes in environmental conditions, TAGs can help the production of polar lipids by transferring a special acyl group to cause a rapid adaptive rearrangement of the membrane [57, 58].

## Approaches to promote lipids production

Various species of microalgae have different type and quantity of lipids [2] but the basal levels can be altered by modifying lipid metabolism in different ways. A biochemical engineering approach is one method based on the manipulation of the nutritional and/or cultivation conditions. The different cultivation conditions include exposure to different wavelengths and light intensity, carbon dioxide levels, temperature, available nutrients [27, 59], stress to heavy metals, stress to salinity [2, 51], and the use of nanoparticles (NPs). Other approach is through genetic engineering, where specific genes, related to lipid metabolism, are manipulated to improve synthesis, storage and structural contents of lipids inside the microalgae cell.

### Effects of light on lipid production and accumulation

Light is one of the most important factors for the development of the microalgae and the production of biomass. More importantly, wavelength and light intensity can cause drastic changes on how the microalgae grow and produce/accumulate lipids. Among the different algal species, photoautotrophic species are the only ones that require light to grow, using light as the energy source for their bioprocesses. However, it is important to study other microalgae species such as the photoheterotrophs and the mixotrophs, to improve biomass, especially since around half of the dry weight of the microalgal biomass is carbon, and lipid production [15, 60].

Within the literature, there are different studies that report on the effect of different light wavelengths on microalgae growth and metabolism. Some reports confirm the effect of blue (400–500 nm) and red (600–700 nm) wavelengths in the development of microalgae and the optimal performance of key enzymes (structural alteration) in photosynthesis processes and product formation [61]. In these cases, cell pigments play an important role in the absorption of specific light wavelengths, for example, the normal range of the photosynthesis spectrum in microalgae cells is 400 nm to 700 nm; therefore the pigments inside the cell (mainly chlorophyll a and b) correlate their adsorption spectrum to the optimal conditions of microalgae growth [62, 63].

Regarding this, Severes et al. reported that a combination of the wavelengths of red and blue light for the lighting of a culture of *Chlorella* sp. can cause an increase in the biomass production. Also, they have shown that the dry weight of lipids contained in *Chlorella* cells is doubled when the wavelength of red light is applied during the growth periods [61]. Similar results were published by Monika and colleagues where they report on the effect of different light wavelengths (light colors: white, red, yellow and green) on the growth behavior and lipid accumulation of a *Chlorella* sp. strain. According to their results, the best growth and the highest lipid accumulation obtained were when the cells are exposed to red light, meanwhile, the minimum values in these two aspects were obtained with the green light [64].

In another study, a *Chlorella vulgaris* strain was grown under different internal LED strips of cold white, blue and red, showing that after 10 days with 18:6 light/dark periods, with wastewater as a culture medium and with an initial cell number of approximately 10^6^ cells/ml they obtained a microalgae cultivated with blue light that reach the highest production of lipid content (34.06%) due to its efficiency and deep penetration [62]. Meanwhile, Osman et al. discover that the color of the cultivation light affects both the total lipid content and the fatty acid profile; they reported that the saturated fatty acids were not different in the blue, white and green lights, but they were reduced with the red light. The treatment also found that the tendency of the saturated fatty acids was the opposite of the monounsaturated fatty acid [65]. The effect of the light on total oil accumulation and lipid composition correlate with reports presented by Markou [66], Liu et al. [67], Marchetti et al. [68] and Wacker et al. [69].

The light intensity is as important parameter as it has been shown to drastically affect the growth of microalgae and their lipid content. Typically, both low light intensity and extremely high light intensity, causes unfavorable growth and undesirable responses in microalgae cells. In many cases these extreme light intensities cause photo-inhibition and photo oxidation that affect lipid content [2, 70]. Identifying the optimum range of light intensity aids in obtaining the optimal growth of microalgae and a higher lipid production [71, 72]. Many research efforts have been made to show the light intensity needed to obtain the maximum growth and lipid content, but it varies with microalgae species. For instance, Pal et al. report that the microalgae *Nannochloropsis* sp. produces the highest lipid amount (47% of dry weight) under a light intensity of 700 μmol photons/m^2^/s [73]. Takeshita et al. showed that *C. sorokiniana*, *C. viscosa*, *C. emersonii*, *C. vulgaris*, *P. beijerinckii*, and *P. kessleri* CCALA255, NIES-2152, and NIES-2159 can produce more lipids with a light intensity of 600 μmol photons/m^2^/s [74]. Further studies indicate that lipid accumulation in *Scenedesmus* sp. was increased 11-fold when the intensity of light changes from 250 to 400 μmol photons/m^2^/s [67], while other study demonstrate that the highest lipid accumulation in a *Ettlia* sp. culture (291,4 mg/L/day) is observed at a light intensity of 1500 μmol photons/m^2^/s [75]. Furthermore, lipid content is not affected in several microalgae species when exposed to changes in light intensity; such is the case of *Scenedesmus obliquus*, where experiments that exposed it to a range of light intensity from 200 to 1500 μmol photons/m^2^/s show that the lipid content remains unchanged [76].

Nowadays, two main categories of microalgae cultivation methods have been suggested. One method involves open systems with configurations of open ponds, tanks, raceway ponds and the other methods involve closed culture configuration systems such as photo bioreactors (PBR) [77, 78]. PBRs are flexible systems that can vary in shape, such as including tubular, vertical columns and flat plates [79]. PBRs have the ability to adjust and have a tighter control in the culture-parameters [80]. Table 1 shows the main advantages and disadvantages of these two types of microalgae cultivation systems [77, 81].

**Table 1:** Main advantages and disadvantages of open pond and closed culture systems for microalgae biomass production

Cultivation system	Advantages	Disadvantages
Open	Low operating costs	Process and contamination controls with low efficiency
	Easy to scale up	High evaporation rate
	Cooling through direct contact with atmosphere	Requires lots of land to produce
	Good gas interchange	Poor light penetration
		High loss of CO_2_
		High harvesting costs
		Low production performance
		Low control over growth factors (e.g. evaporation, temperature)
PBR	Lower contamination risk	High operating costs
	High production performance	High construction cost
	Relatively low harvesting cost	Difficult to scale up
	High light use efficiency	
	Requires low land to produce	
	High control parameters of culture	
	Low loss of CO_2_	

Both open and PBR cultivation systems are used in large-scale commercial production of biomass to obtain different chemical compounds, such as fatty acids, proteins, anti-oxidants, pigments and animal feedstock [82]. Nevertheless, considering the sensitivity of the open pond system to contamination, the open pond systems is mainly used for biomass production of microalgae strains, which grow in specific conditions, such as high nutrient concentration. Jorquera et al. introduced *Chlorella* sp., *Spirulina* sp. and *Dunaliella salina* as strains with a requirement of specific growth condition [83]. Hence, it is indispensable to employ PBRs to produce high-value products such as human nutrient and pharmaceutical products from microalgae which are grown under tightly controlled conditions [84].

One aspect to be considered is the difficulty of controlling light exposure in an industrial production setting. One main design aspect of culture systems involves the ratio between light exposure surface area and volume of culture. The literature shows that typically high ratios between these two parameters lead to higher biomass yields and growth in microalgae. Therefore there is an intense area or research being developed to design innovative culture systems with the purpose of maximizing light exposure area per volume of culture. Although both open pond and closed PBRs can use either natural sunlight or artificial illumination for microalgae cultivation [85], closed PBRs offer better control of light than open systems. Controlling wavelength and intensity of light is more feasible in a lab-scale PBRs compared to industrial settings. Therefore, optimal conditions are determined in a lab-scale PBR to further test large-scale models. One of the complexities in the scalability of these systems is correlating light permeation into the cultures system since it decreases exponentially with distance from the light source, posing a difficult engineering problem when scaling PBR systems [86, 87].

Contrary to closed PBRs, surface-to-volume ratio and the corresponding light penetration in open ponds system are far from ideal; although direct sunlight is too strong for most microalgae, most of them only need 1/10 of direct sunlight [88]. Mainly, in most open pond system, only the top 7–10 cm of water are exposed to enough light penetration for efficient photosynthesis [89], the causes are bulk algal biomass that is accumulated in the surface which leads to blocking natural light sources and preventing it from reaching deeper into the water [90]. However, one main advantage of open culture systems is that they are inexpensive to install and operate [87].

When comparing both open and closed systems, choosing between them heavily depends on specific conditions of the user, however, it should be clear that for research and in order to expand understanding the behavior of a microalgae culture, the most convenient way is through PBRs. In this regard, a simulation model for algae production in greenhouses system, developed by Hermans et al. [91] and then adopted in industrial settings, was used to calculate the yearly algae production in a one-hectare greenhouse filled with PBRs in the Netherlands [92]. Their study showed that the minimum cost price of algae produced in a one-phase and two-phase tubular PBR were between 16–35 € and 11–19 € per kg of dry algae biomass, respectively. Very interestingly the report included depreciation of the PBR and greenhouse equipment, labor, maintenance and electricity required for illumination, this last one accounting for the highest percentage of the total cost.

### Carbon dioxide

The atmosphere, industries discharge gases and carbonate salts are highly valuable sources for microalgae cultures and other photosynthetic microorganisms, to capture CO_2_ and in the presence of sunlight use the inorganic carbon to produce biomass and chemical compounds of interest [93] (Table 2). For microalgae to grow and be metabolically active, there are specific maximum and minimum CO_2_ level parameters that must be reached. These optimal CO_2_ levels influence lipid production and their accumulation within the cell [2]. Although the increase of CO_2_ could help in the production and accumulation of lipids in the microalgae cells, as mentioned previously there is also a maximum level where the excess CO_2_ levels leads to disruptive effects in lipid production and cell growth. Previously it was believed that the microalgae cells develop best at high concentrations of CO_2_ and this was corroborated with experiments performed with some species of *Nannochloropsis*, which are not inhibited at high CO_2_ levels. However, later experiments have demonstrated that most species exhibit a maximum range where CO_2_ become non-optimal and in some cases lethal to the culturing of microalgae [94–96].

**Table 2:** Studies on the effect of CO_2_ levels on the accumulation of lipids in different species of microalgae

Microalgae strain	Used CO_2_ concentration	Change of lipid amount	Refs.
*Chlorella* sp. BTA 9031	3% (v/v)	Accumulated 25% of lipid as a percentage of dry cell weight	[98]
*Chlamydomonas* sp. JSC4	4% (v/v)	Generated maximum lipid content (65.3%) and productivity (169.1 mg/L/day)	[99]
*Chlorococcum littorale*	5% (v/v)	Lipid content increased up to 34% wt	[100]
*Scenedesmus obliquus* CNW-N	The optimal CO2 consumption rate was 1420.6 mg/L/day	The highest productivity of lipid (140.35 mg/L/day) is achieved	[101]
*Synechocystis* sp. PCC6803	3% (v/v)	The total lipid content increased up to 14% of dry weight	[102]
*Porosira glacialis*	20–25% levels of CO_2_	The total lipid content increased from 8.91 to 10.57% in cell dry mass Docosahexaenoic acid content increased from 3.90 to 5.75% EPA decreased from 26.59 to 23.66%	[103]
*Attheya longicornis*	20–25% levels of CO_2_	Did not show any significant increase in total lipid content	[103]
*Nannochloropsis oculata*	3% (v/v)	Demonstrated high lipid content (53.2 wt%)	[104]
*Scenedesmus* sp.	10% CO_2_	Lipid productivity reached up to 20.65 mg/L/day	[105]
*Chlorella vulgaris*	30% CO_2_	The highest lipid content (45.68%) and lipid productivity (86.03 mg/L/day) is obtained	[106]

When a culture of microalgae cells is aerated with high concentrations of CO_2_, a part of the carbon is used by the cells for the process of photosynthesis, but the remaining carbon could be converted to carbonic acid (H_2_CO_3_). This compound can cause acidification of the medium, altering cell growth and metabolic pathways. Ying et al. studied pH changes in the medium when exposing the culture to different CO_2_ aeration parameters, and they report that dramatic pH changes that lead to damage of enzymes involved in the photosynthesis process. Hence, it is very important to consider optimum pH levels for the various species of microalgae to determine the CO_2_ exposure optimal for biomass growth and lipid production and accumulation in the cells [97].

A variety of works in the literature have focused on understanding the effect of different CO_2_ concentrations on microalgae cells. Montoya et al. determined a high concentration of fatty acids and lipid productivity (29.5 mg/L/day) in a culture of *C. vulgaris* with a CO_2_ concentration of 8% (v/v) [107]. In another work, Ying et al. reported an inhibition of growth in a culture of *D. Salina* with a 0.02 mol CO_2_/L concentration and discover that if the concentration increases more than 0.02 mol CO_2_/L (i.e. constant doses of 50% (v/v) CO_2_) it turned out to be deadly for its growth [97]. Moreover, *Chlorella Pyrenoidosa* an oleaginous specie, was cultivated under 5% (v/v) of CO_2_ concentration, obtaining the highest lipid productivity, 107 mg/L/day. Bagchi and Mallick reported that a cultivated *Scenedesmus Obliquus* (Turpin) Kützing GA 45 with 15% (v/v) of CO_2_ concentration was able to obtain a lipid accumulation performance of 850 mg/L in a period of 16 days [108]. Finally, green microalgae of the *Chlamydomonas* sp. JSC4 strain were cultured under 4% (v/v) CO_2_ achieving a maximum lipid productivity (169.1 mg/L/day) [99].

### Temperature

Temperature is another important factor that affects growth and the lipid content in microalgae cells. Different studies have shown that temperature changes have a crucial effect on microalgae development, lipid production and formation of fatty acids, however optimal parameters depend on the species. The literature shows that the biochemical pathways related to the synthesis and accumulation of lipids are controlled by enzymes with a high sensitivity to thermal variations [109]. Some researches based on reported results suggest that as the temperature increases, microalgae tend to produce and accumulate saturated fatty acids; opposite to this, at low temperatures microalgae tend to produce and accumulate unsaturated fatty acids. Menegol et al. studied the effects of temperature on *Heterochlorella luteoviridis*, finding that with a temperature set at 22 °C, it could get a 40.7% of PUFAs (of the total percentage of fatty acids), and when the temperature change from 22 to 27 °C, the percentage of saturated fatty acids was increased (52.9%) [110].

Depending on the objective of the study, the ideal temperature for microalgae cultivation can be selected, but as with the other parameters, the optimal temperature will vary for each species of microalgae affecting significantly their growth and lipid production. Such is the case observed with *Nannochloropsis limnetica*, where the cells can grow in the range of 15 °C to 27 °C but their maximum growth and lipid productivity occurs at 22 °C [111]. Converti et al. have studied *N. oculata* and *C. vulgaris*, they suggest that an increase in temperature causes a decrease in lipid content in *C. vulgaris,* and the decrease causes an increase of lipid content. Meanwhile, in *N. oculata*, increase and decrease temperature resulted in increase and decrease of lipid production, respectively [112].

Two strains of *Monoraphidium consortiums* and *Desmodesmus quadricauda* showed that a decrease in temperature of up to 13 °C provides an optimal condition for lipid accumulation [113]. In a different study, the optimal temperature for *Tetraselmis subcordiformis* and *N. oculata* was 20 °C and 30 °C, respectively [114]. In addition, considering that there are open and closed systems, regulation for high and low temperatures can only be carried out in closed PBRs, since open systems are typically operated at environmental conditions with constantly varying temperature [51]. Temperature is a very important condition that should be considered in the cultivation of microalgae, a closed system is an excellent method for temperature control, especially when an optimal temperature has been determined to obtain the best lipid productivity and biomass quantity.

### Nutrient limitation

Essential inorganic nutrients such as nitrogen, sulfur, carbon, iron and phosphorus have remarkable impact on growth, reproduction and metabolism of microalgae cells. Nutrient limitation is an applied and promising strategy used by many researchers to change and control the microalgae cell cycle and the biochemical pathways linked to lipid production and accumulation. The lack of nutrients causes undesirable situations inside the cell, generating the accumulation of more lipid compounds as a response, this technique has been therefore exploited by researchers and the industry to increase lipid production and accumulation [115].

In a culture medium, cell growth is linked to availability of a high concentration of nutrients in the culture, especially during the early life cycle stages of cell growth; a rich media therefore leads to a maximization of biomass productivity. Then, after reaching the necessary biomass, nutrient limitation can cause an environment of stress and a ramp-up in lipid production, especially observed in the late growth-stages. Most of the work and studies have shown that numerous species of microalgae produce and accumulate higher amount of lipids, specially the TAGs, in nitrogen-limited mixotrophic conditions [116–119]. However, it is possible that nutrient limitation might affect other biochemical pathways in the cells impacting indirectly lipid productivity [120].

Several studies have explored different nutrient limitation techniques in different strains to understand and optimize different output parameters. Yang et al. showed that under situations of phosphorus or nitrogen deficiency the fatty acid yield in *Chlamydomonas reinhardtii* is significantly increased [121]. Cordeiro et al. carried out a study of the effects of phosphorus and nitrogen levels on the growth of species of *Microcystis*, they reported that the obtained lipid performance from *Microcystis panniformis* and *Microcystis novacekii* had the inverse and direct correlation with nitrogen (35.8%) and phosphorus concentration (31.7%), respectively. They also have reported that *Microcystis aeruginosa* had an inverse correlation with the nutrient concentration (23.3%) [122]. Furthermore, Mata et al. showed that by increasing ten times the concentration of nitrogen in the culture medium, lipid productivity and content increased 33.5% and 47.4 mg/L/day, respectively in *Dunaliella tertiolecta* [123]. It was also shown that by increasing the iron concentration 10 times (compared to the standard culture medium), the lipid productivity increased from 14.6 mg/L/day to 28.0 mg/L/day. Finally, Figueroa et al. cultivated *C. reinhardtii* under limited mixotrophic conditions, and had a significant 66% increase in lipid production (0.08 g/L).

### Heavy metal stress

There are some heavy metals such as Cu, Fe, Mn, Ni and Zn that are vital micronutrients in many biological processes [124–126] since they have essential roles as precursors of vitamins, catalytic cofactors for numerous metallo-enzymes, and structure proteins of the cell membrane [124, 127, 128].

Microalgae have proven to be efficient and effective in the removal of heavy metals and are able to tolerate high concentrations of heavy metals through different mechanisms such as coupling metals to surface proteins, expression of cellular ligands to create metal complexes, triggering of efflux pumps to excrete metal ions, and kidnapping heavy metals through polyphosphates, phytochelatins and metallothioneins [129]. The accumulation of high concentrations of heavy metals can cause the production of reactive oxygen species (ROS) [130], inhibition in the synthesis of chlorophyll [131] and negative disruption on cell proliferation [132] affecting lipid accumulation within the microalgae cell [133]. In different studies, heavy metal stress has led to increase the lipid content in some microalgae [134].

Ren et al. evaluated the effects of Fe^3+^ (0–0.12 g/L), Mg^2+^ (0–0.73 g/L) and Ca^2+^ (0–0.98 g/L) on lipid accumulation in *Scenedesmus* sp. cells and suggested that the total lipid content and lipid productivity increased up to 28.2% and 29.7%, respectively with the addition of EDTA during cultivation [133]; this implies that, the main metabolic pathways related to lipid synthesis and breakdown in *Scenedesmus* sp. cells could be modified by Fe^3+^, Mg^2+^ and Ca^2+^ [135]. These metal ions have multiple physiological functions that affect metabolic activity of microalgae cells and their lipid accumulation processes [136], for example, Ca^2+^ is a universal messenger involved in the signaling of environmental and developmental stimuli [137]. It is worth noting that Mg^2+^ has been recognized as an important signaling ion, both activating and mediating many biochemical reactions, such as regulation of carbon fixation in chloroplasts in the Calvin cycle [138, 139]. In addition, the literature shows that the increment of

Mg^2+^ could assist Acetyl-CoA carboxylase (key regulator of fatty acid synthesis) function to increase the neutral lipid content in microalgae cells [136]. Battah et al. examined the effect of heavy metals (Mn^2+^ and Co^2+^) on the lipid content of *C. vulgaris* [140], using manganese chloride (MnCl_2_) at a concentration of 2 μM, 10 μM and 12 μM. The results demonstrate that all of these concentrations increased lipid content significantly by 14%, 16% and 15%, respectively. They also discovered that if cobalt nitrate is added at different concentrations, lipid content can be increased up to 25% more, compared to the corresponding controls.

An additional study reports up to 56.6% increase in the total lipid content in *C. vulgaris* at 5 different Fe^3+^ concentrations in the culture medium [135]. Einicker-Lamas et al. mentioned that cadmium increases the total lipid content in *Euglena gracilis*. For the case of cadmium and its effects on *C. vulgaris*, reports have shown that TAGs, acetone mobile polar lipids (AMPL) and phospholipids (PL) were the main lipid classes after exposing *C. vulgaris* to different combinations of cadmium (2 × 10^−8^; 10^−7^ M) and nitrogen (2.9 × 10^−6^ to 1.1 × 10^−3^ M) Furthermore, by changing the combination of nitrogen and cadmium in the medium it is possible to alter and control lipid composition [134].

### Nanoparticles

Researches have used various types of metallic NPs, within a range of 5–100 nm, since they exhibit different physical and chemical properties than the same metals at the macroscale [109, 141]. The diverse physicochemical behavior of metallic nanoparticles have allowed their use for many different applications in drug delivery systems, the food industry, cosmetics, optics and the synthesis of multifunctional biomaterials [142]. One very recent application of NPs is linked to their ability to improve gas–liquid mass transfer rate in fermentations [143, 144]. The presence of the NPs improves the mass transfer coefficient at the gas–liquid interface [145]; therefore, the assumption is that the increase of CO_2_ concentrations through NPs can affect the growth rate and the induction of lipids in some microalgae.

Jeon et al. used silica nanoparticles and methyl-functionalized silica (SiO_2_–CH_3_) nanoparticles in a *C. vulgaris* culture. They used Blue-Green medium (BG-11) and grew microalgae that used solely CO_2_ as a carbon source; they were able to observe that the NPs increased the gas–liquid mass transfer rate in this CO_2_/medium culture system and improved both growth and lipid accumulation in the cultivated microalgae. They reported that the use of both NPs causes an increase in the volumetric mass transfer coefficient (k_L_a) of 31% and 145%, respectively; the results also showed that, although the addition of silicon NPs leads to an increase in cellular dry weight and in fatty acid methyl ester productivity, the highest cellular dry weight (1.49 g/L) and the highest fatty acid methyl ester productivity (610%) were obtained by the addition of 0.2 wt% SiO_2_–CH_3_ NPs [146].

Similarly, Ahn et al. examined the effect of magnetic cobalt ferrite/silica NPs and methyl functionalized magnetic silica NPs (methyl-MSNs) on growth and lipid production in a culture of *C. vulgaris* by improving gas–water mass transfer and increasing the concentration of dissolved CO_2_. Reporting that for the k_L_a of 0.3 wt% of the MSNs and methyl-MSNs were 3.11/h and 4.01/h, respectively; and the use of 0.3 wt% Methyl-MSNs yielded the highest mass transfer rate [147]. Nonetheless, improving the rate of mass transfer not only does not increase the lipid content, but also decreases it sharply (to 3.37% and 4.57%, respectively).

It has been found that some metallic NPs such as Ag, Au, CuO, ZnO, Se, Pd and FeO turn out to be highly toxic for different organisms [148–153]. One of these affected organisms is microalgae (Table 3); the toxic effect of NPs is related to ROS production and the induction of oxidative stress, this is only achieved when the concentration of NPs reaches an effective level [149–151].

**Table 3:** Toxic effect of different NPs in several species of microalgae

Microalgae strain	Type of NPs	Size of NPs	Used concentration	Refs.
*Platymonas subcordiforus* *Chaetoceros curvisetus* *Skeletonema costatum*	Co NPs	30 nm	67.2 mg/L 38.6 mg/L 21.5 mg/L	[154]
Soil alga *Chlamydomonas reinhardtii*	Ag NPs	< 100 nm	0–50 mg Ag NPs/kg dry weight soil	[155]
*Chlorococcum sp.* *Scenedesmus rubescens* *Dunaliella tertiolecta* *Tetraselmis suesica*	ZnO NPs	< 100 nm	0.081–810 mg/L	[156]
*Navicula sp.* *Chetoceros* sp.	CoO NPs	< 50 nm	2 mg/ml	[1]
*Dunaliella salina*	SiO_2_ NPs	11–14	0.1, 0.3, 0.85, 2.4, 7, 20 and 50 mg/L	[157]
*Chlorella vulgaris* (KCTCAG10002)	ZnO NPs CuO NPs NiO NPs TiO_2_ NPs Fe_2_O_3_ NPs	40–100 nm 30–50 nm 30 nm < 25 nm 20–40 nm	8, 16, 33 mg/L 0.5, 1, 2 mg/L 4, 9, 18 mg/L 20, 40, 80 mg/L 22, 45, 90 mg/L	[158]
*Dunaliella salina*	Al_2_O_3_ NPs	20 nm	0.005, 0.026, 0.14, 0.7, and 3.8 mg/L	[159]

Some researchers mention that if microalgae are exposed to adequate doses of NPs, they can induce oxidative stress and thus improve lipid production [142, 160, 161]. He et al. evaluated the effects of Carbon nanotubes (CNTs), α-Fe_2_O_3_ NPs and MgO NPs on lipid production of *Scenedesmus obliquus*, and they discovered that exposure to 5 mg/L CNTs, 5 mg/L Fe_2_O_3_ and 40 mg/L MgO NPs increased the lipid content up to 8.9%, 39.6% and 18.5%, respectively. In addition, when microalgae were exposed to high doses of NPs, biomass and lipid production decreased, due to the high concentrations of ROS generated that caused cell death [142].

Similarly, Kang et al. used the oxidative stress of TiO_2_ NPs to stimulate and enhance lipid productivity in *C. vulgaris* UTEX 265 and suggested that oxidative stress causes the accumulation and decomposition of lipid productivity. They also mention that the highest productivity of fatty acid methyl ester (18.2 g/L/day) was obtained with low doses of TiO_2_ NPs (0.1 g/L) and a short induction time of 2 days [160].

Other reports related to the use of NPs to improve lipid productivity are listed in Table 4. It should be emphasized that the use of NPs for the improvement of lipid productivity is a unique method and has some disadvantages for example, how expensive it is to recycle NPs for the following experiments. Therefore, more in depth studies should be carried out to describe in detail the stability and environmental effects that NPs can cause.

**Table 4:** Improvement of lipid productivity using different types of NPs in some species of microalgae

Microalgae strain	Type of NPs	Utilization	Lipid profile change	Refs.
*Chlorella vulgaris*	Cu NPs	Metal resistance induction	Total lipid increase (up to 32%)	[162]
	Mg NPs	Photosynthesis enhancement	Lipid content increase (0.43 mg/L)	
	Zn NPs	Metal resistance induction	Total lipid content increase (0.74 mg/L)	
	Pb NPs	Increase of growth rate	Total lipid content increase (0.76 mg/L)	
*Isochrysis galbana*	Fe NPs	–	No significant difference in total lipid content	[163]
*Pavlova lutheri*		Increase of growth rate	Increase of the total lipid (up to 12 pg/cell)	
*Tetraselmis suecica*		Increase of growth rate	Increase of the total lipid (up to 40 pg/cell)	
*Chlorella vulgaris*	Nanoscale MgSO4	Photosynthesis enhancement	185.29 ± 4.53% improvement in lipid production	[164]
*Chlorella* sp. KR-1	CTAB-decorated Fe3O4 NPs	Improvement of harvesting and cell disruption efficiency	The cells harvested using CTAB-OTES-MNP yielded an approximately 2.3-fold-higher lipid content compared with the control extracted by only hexane	[165]
*Nannochloropsis maritima*	Fe3O4 NPs	Improvement of harvesting efficiency	The algal biomass increased up to 1.02 g/L at day 18 (subsequently, more total lipid amount is achieved)	[166]

### Saline stress

Salts play a vital role in the physiological and bio-chemical pathways of growth, reproduction and metabolism of fatty acids in microalgae, therefore, saline stress is one of the most efficient enrichment strategies for lipid content. Because of this, many researchers have focused on studying salt stress for this purpose [167–169].

Saline stress is known to cause a difference in osmotic pressure within microalgae cells, which, generates a stress-response that leads to the modification of their metabolism which will allow the microalgae to adapt to these new conditions [170, 171]. Changes at the metabolic level causes saline fluctuations within the cell, increasing significantly the lipid content; it has even been found that variations in the concentration of salt in the growth medium not only increase the total lipids of the microalgae cells, but can also altered lipid composition [51].

Bartley et al. investigated the effects of salt stress on the growth of marine microalgae *Nannochloropsis salina.* They grew it at 22 PSU (particle salinity unit) until the culture reaches the stationary phase and then they increased the concentration of salts to 34, 46, and 58 PSU. They reported that the lipid content increased significantly under these salt concentrations, obtaining the highest total content of fatty acids (36% dry tissue mass) at 34 PSU [172].

Meanwhile in a study by Salama et al*., Chlamydomonas Mexicana* and *Scenedesmus obliquus* were grown in a culture medium with different levels of salt stress reaching up to 100 mM NaCl; showing that the maximum lipid content obtained (37% and 34% respectively) from *C. Mexicana* and *S. obliquus* were achieved with a concentration of 25 mM NaCl. They also investigated the composition of fatty acids, finding that linoleic acids (41%) and oleic acids (41%) were the dominant fractions. Although the data on the effect of NaCl on the fatty acid composition of microalgae lipids are scarce and conflicting, these results also show that the higher concentrations of NaCl in some species of microalgae such as *Chlamydomonas Mexicana* and *Scenedesmus obliquus* can improve the composition of their fatty acids [173]. Depending on the type of lipid used, different levels of NaCl can be used to alter the fatty acid composition. In other words, depending on which one of polyunsaturated fatty acids, monounsaturated fatty acids and saturated fatty acids are needed, the suitable NaCl level can be used.

Pandit et al. grew two strains of microalgae (*C. vulgaris* and *Acutodesmus obliquus*) in a medium that contained different levels of salt concentration (from 0.06 to 0.4 M NaCl) and they reported that the maximum amount of lipids (49% and 43%, respectively) was obtained at a concentration of 0.4 M NaCl [174]. Besides, *Acutodesmus dimorphus* showed a significant accumulation of lipids (33.40 ± 2.29%) in 200 mM NaCl of added medium; and the lipid accumulation increased significantly up to 43%, when saline stress extended to 3 days [175].

The type of salt used to cause the saline stress also has an effect on the accumulation of lipids in microalgae. Srivastava et al. cultivated *Chlorella sorokiniana* CG12(KR905186) and *Desmodesmus* GS12(KR905187) with different types of salts (NaCl, KCl, MgCl_2_ and CaCl_2_) and found that with CaCl_2_ the maximum effect on lipid production was obtained, improving up to a 40.02% and 44.97% in *CG12* and *GS12*, respectively. It is assumed that Ca^2+^ plays a definitive role in cell signaling under conditions of salt stress which causes an increase in the synthesis of lipid compounds [176].

### Genetic modification of microalgae to increase lipid production

Employing molecular biology to genetically alter microalgae is an approach that offers an alternative to obtain better lipid productivity. Recently, different genetic engineering methods have received a lot of attention from researchers because they are considered novel and especially tunable tools [177, 178]. In general, it seeks to reduce, inhibit or over express one or several genes related to the production of a metabolite of interest. For the case of the microalgae, these genes are related with the photosynthetic process, the growth rate, improved resistance against extreme conditions such as pH, salinity, temperature and genes that have great importance in the metabolism of lipids [109]. The impact on the microalgae are related to: fast growth and large cell size for high biomass production, high lipid yield, the ability to secret lipid into media, adaptive capability to environmental fluctuations and stress and the ability to form flocs for easy and low-cost harvesting [194].

However, one of the main limitations of this approach relies on the data available to do such modifications; sequencing the genomes of microalgae and having them available facilitates genetic manipulation, allowing to know with greater detail and precision the different genes that participate in the different metabolic pathways. Several nuclear microalgae genomes have been sequenced (*C. reinhardtii*, *P. tricornutum*, *T. pseudonana*, *Cyanidioschyzon merolae*, *Ostreococcus lucimarinus*, *Ostreococcus tauri*, and *Micromonas pusilla*). However there are a lot of ongoing projects to have more genomes available [181, 184]. Despite this, it is estimated that there are 72.500 species of microalgae but only about 44.000 have been described [186].

For genetic modification of microalgae there are a variety of bioengineering methods that can be applied: Random Mutagenesis, Clustered Regularly Interspaced Short Palindromic Repeats—CRISPR associated with the protein 9 (CRISPR–Cas9), Transcription Activator-Like Effector Nucleases (TALEN) and Zinc-Finger Nucleases (ZFN) used mainly for the alteration of the gene sequence [179–181]; while the use of micro RNA (miRNA), short interfering RNA (siRNA) and homologous recombination allows the activation and repression of genetic expression [182–184]; meanwhile agitation in the presence of glass bread or silicon, carbide whiskers, electroporation, biolistic microparticle bombardment and *Agrobacterium tumefaciens*-mediated gene transfer has been used to transfer DNA into microalgal cells [4, 181]. The efficiency of transformation strongly depends on the microalgae specie and both the genetic modification method and the transformation method must to be carefully selected according to the specie and type of modification.

The use of the CRISPR–Cas9 system allows the regulation of the expression of multiple target genes [185], the expression of complex traits through the multigene engineering. Since 2014, the use of this tool marked a beginning of a new age of genome editing in microalgae; although the main challenge of using this tool is the toxicity of the Cas9 nuclease (with a mutation rate of 10%); which has an alternative, the use of ribonucleoproteins [4, 188, 189].

The majority of the genetic edition reports on microalgae for the increase of lipid production have been carried out in the study models as *Chlamydomonas* and *Chlorella* [186]. The earliest successful DNA modification was accomplished by Rochaix and Van Dillewijin in *C. reinhadtii* [4, 187]. And in the case of fatty acid biosynthesis, Roessler isolate the acetyl-CoA carboxylase in 1990 to later transform the diatoms *Cyclotella cryptica* and *Navicula saprophila* [51, 193].

One of the first experimental reports related to this was done by Dunahay and colleagues when they tried to introduce additional copies of the acetyl-CoA carboxylase gene in the diatom *Cyclotella cryptica* to manipulate the lipid accumulation [187]. Kang et al. investigated the gene of a Wrinkled1 transcription factor type AP2 in *Arabidopsis thaliana* (AtWRI), whose main function is to regulate lipid biosynthesis in plants, and they transferred it in the microalgae *Nannochloropsis salina* [188]. The characterization of the transformed cells revealed that the total lipid content increased by 36.5% compared to the wild-type strain.

One of the best methods in the genetic engineering approach uses the RNA silencing technique. Deng et al. investigated the CrCO gene of *C. reinhardtii* [189], a homologous gene of the circadian-regulated CONSTANS gene (CO) which plays an important role in the photoperiod and flowering time [190, 191]. They determined that the repression and overexpression of the CrCO gene can change lipid accumulation in microalgae cells and the silencing of the gene (by RNA interference, RNAi) can increase the lipid content and the levels of TAGs up to 24%. Trentacoste et al. reported that the gene knockout of a multifunctional lipase/phospholipase/acyltransferase increased the amount of lipids in the cell without affecting the growth of the *T. pseudonana* diatom [192]. In addition, they discovered that antisense-mediated knockout mutants of the diatom had 3.3 times more lipid content than the wild-type variants in the exponential phase of growth.

There are many other experiments and reports based on the genetic engineering methods applied to the various species of microalgae (Table 5). However, these methods have some limitations: high production cost, low growth rate, low transformation success and incomplete genetic and characterization problems for the scaling of microalgae culture [51, 193, 194].

**Table 5:** Improvement of lipid content in different species of microalgae with genetic engineering methods

Microalgae species or strain	Type of modification	Changes in lipid profile in the microalgal cells	Refs.
*Chlamydomonas reinhardtii*	Repression of Major lipid droplet protein (MLDP) gene expression	40% increase in the average lipid droplet diameter	[197]
*Chlamydomonas reinhardtii*	Knockout of citrate synthase gene	TAG level increased up to 169.5%	[198]
*Chlamydomonas reinhardtii*	Artificial silencing of *Diacylglycerol acyltransferase 2*–*4* gene (CrDGAT2-4)	24%-34% increase in lipid content	[199]
*Chlamydomonas reinhardtii* (starchless mutant)	Inactivation of Adenosine diphosphoglucose pyrophosphorylase (ADP-glucose pyrophosphorylase)	10-fold increase in TAG	[200]
*Chlorella minutissima* UTEX 2219	Overexpression of glycerol-3-phosphate aceyltransferease gene, lysophosphatidic acetyltransferase gene and diglyceride acyltransferase	2-fold increase in lipid content	[201]
*Scenedesmus obliquus* (starchless mutant)	Knockdown of competitive pathways genes	Increase in TAG accumulation of up to 51%	[202]
*Phaeodactylum tricornutum*	Heterologous gene expression of *acyl*–acyl carrier protein thioesterases (Acyl-ACP thioesterases)	Increased accumulation of shorter chain length fatty acids	[203]
*Synechocystis* sp.	Cyanophycin synthetase gene deletion	Fatty acids secretion into the medium	[204]
*Synechocystis* sp.	Phosphotransacetylase gene deletion	Increase in production of fatty acids	[204]
*Phaeodactylum tricornutum*	Suppression of TAG lipase gene expression	Increase of the lipid content (0.04 ± 0.01 mg TAG/mg dry weight)	[205]
*Scenedesmus obliquus*	Successful expression of diacylglycerol acyl-transferase gene	Enhanced 128% of lipid content.	[190]
*C. reinhardtii*	Knock-down of PEPC enzyme with CRISPRi	Enhanced lipid production up to 94%	[191]
*P. tricornutum*	Expression of malic enzyme	Enhanced lipid productivity by 2.5 in comparison with wild-type	[192]
*P. tricornutum*	Overexpression of glucose-6-phosphate dehydrogenase (G6PD)	Increased production of lipids up to 55.7% of dry weight	[186]
*T. pseudonana*	Knock-down of a multifunctional lipase/phospholipase/acetyltransferase enzyme	Mutant strains produced 2.4- to 3.3-fold higher amounts of lipids in comparison with wild-type	[186]

One problem of working with genetically modified (GM) microalgae is the environmental impacts and the ethics regarding their release. It is worthy to note that, the intentional release of GM organisms such as microalgae into the natural environment must be thoroughly analyzed and accepted by different international committees of experts; since in the error or consensual release of GM microalgae, they can remain in the natural habitat but even reproduce and spread further [195]. This concern related to GM microalgae and the impact to the environment and human health needs to be check by the negative ecological effects like change of food webs structure, displacing native species such as phytoplankton, causing local extinctions, detrimental algal blooms formation, and having serious societal, cultural and economic effects where different toxic strains are involved [196]; in order to make a final decision about their release.

Other examples related to genetic modifications of microalgae to improve resistance and that could be useful to improve lipid content/productivity: one was carried out by Nakamoto and colleagues [194, 195], the small heat shock protein (ch-sHSP) was overexpressed in *Synechococcus elongates* resulting in higher thermo tolerance under light condition (in comparison with wild-type). Schroda and collaborators [194] overexpressed HSP70B in *Chlamydomonas* and find that exhibited greater photosynthetic efficiency by protecting the photosystem II. Finally, Li and colleagues [197] overexpressed the homogentisate phytyltransferase vitamin E2 (VTE2) obtaining a higher protection against oxidative stress.

## Conclusion

Microalgae as unicellular photosynthetic microorganisms can produce high amounts of lipids, which makes them a promise for biofuel production in the sustainable energy sector. Most importantly the growth of microalgae, their maintenance, the extraction of lipids and their subsequent conversion to biofuels must be profitable and competitive with fossil fuels. This review has shown different environmental and genetic engineering strategies that have been explored in order to achieve increased lipid production in different microalgae species and therefore an economically feasible strategy of energy production. These strategies can be used alone or in combination, however, it is necessary to carry out more studies. It is also important to remember that the effectiveness of the strategies and their results will depend on the species, the lipid production objective, the experimental facilities available and the economic resources accessible to the development of the project.

## Data Availability

The datasets used and/or analyzed during the current study are available from the corresponding author on reasonable request.
